# Teledidactic Versus Hands-on Teaching of Abdominal, Thoracic, and Thyroid Ultrasound—The TELUS II Study

**DOI:** 10.1007/s11606-024-08760-4

**Published:** 2024-04-12

**Authors:** E. Höhne, F. Recker, P. Brossart, V. S. Schäfer

**Affiliations:** 1 Department of Rheumatology and Clinical Immunology, Clinic of Internal Medicine III, University Hospital Bonn, Bonn, Germany; 2 Department of Obstetrics and Prenatal Medicine, University Hospital Bonn, Bonn, Germany

## Abstract

**Background:**

The worldwide COVID-19 pandemic has initiated a change in medical education and the development of new teaching concepts has become inevitable to maintain adequate training.

**Objective:**

This pilot study aims to compare teledidactic teaching with traditional face-to-face teaching for abdominal, thoracic, and thyroid ultrasound.

**Design:**

Concurrently, a teledidactic and a face-to-face ultrasound course were held. The students completed seven 90-min modules using mobile ultrasound probes (Butterfly IQ). Each module consisted of a lecture, a demonstration of probe guidance, and independent training.

**Participants:**

A total of thirty medical students took part in the study and were randomly assigned to a teledidactic and a face-to-face group.

**Main Measures:**

An objective structured assessment of ultrasound skills (OSAUS) was performed as a pre-test and as the final exam and ultrasound images obtained during the exam were evaluated using the brightness mode quality ultrasound imaging examination (B-QUIET) scale.

**Key Results:**

No significant difference between the two cohorts on the OSAUS final exam was shown (*p* > 0.05 in all modules). There was a significant difference in the assessment of the images in the focused assessment with sonography for trauma (FAST) (*p* 0.015) and aorta (*p* 0.017) modules. Students in the teledidactic group performed better in both modules, scoring 33.59 (± 2.61) out of 44 in the module FAST (face-to-face group 30.95 (± 1.76)) and aortic images averaged 35.41 (± 2.61) points (face-to-face group 32.35 (± 3.08)).

**Conclusions:**

A teledidactic course for abdominal and thoracic ultrasound examinations is equally effective to traditional face-to-face teaching in this pilot study. Digital implementation with a portable ultrasound machine could be a great opportunity to promote ultrasound education worldwide and over great distances.

## INTRODUCTION

The integration of ultrasound into medical education has not yet been adequately addressed. There is no international consensus on how ultrasound teaching should be incorporated into the traditional curriculum or the way skills testing should be conducted. Accordingly, the extent to which they teach this skill to students has been left to faculties so far and a survey revealed that ultrasound is not taught in the pre-clinical curriculum at the majority of European universities.^1^ While many authors describe the advantage of including ultrasound in anatomy teaching,^2–4^ a systematic review asserts that the value of clinical ultrasound for medical students has not yet been proven and educators should consider whether the financial and time investment involved is warranted.^5^ Besides the lack of guidelines and agreement on an approach to integrate ultrasound, the global COVID-19 pandemic has led to a shift in medical education and has countered previous efforts to provide hands-on teaching. During the last 2 years, new teaching concepts have been established, as traditional face-to-face teaching could not take place as usual in medical education.^6^ The development of new teaching approaches for ultrasound was necessary to guarantee sufficient training.^7^ Attempts have been made to use social media–based networks^8^ or online modules^9^ as well as simulators^10^ to educate ultrasound skills via teledidactic teaching. A previous study demonstrated the feasibility and efficiency of self-learning and telepresence instruction for focused cardiac ultrasound among medical students.^11^ Online teaching has become increasingly important for training, benefiting both medical students and practicing physicians. It has demonstrated comparable effectiveness to face-to-face teaching, as evidenced in studies on sonographic pneumothorax detection for anesthesiologists^12^ and vascular access training for emergency physicians.^13^ It is debatable whether these methods should not be widely used or at a minimum be added to prevent future educational discontinuities.^14,15^ This pilot study aims to conduct a randomized controlled trial comparing the effectiveness of teledidactic lessons and traditional face-to-face teaching in ultrasound instruction for the abdomen, thorax, and thyroid gland.

## METHODS

**Figure 1:**
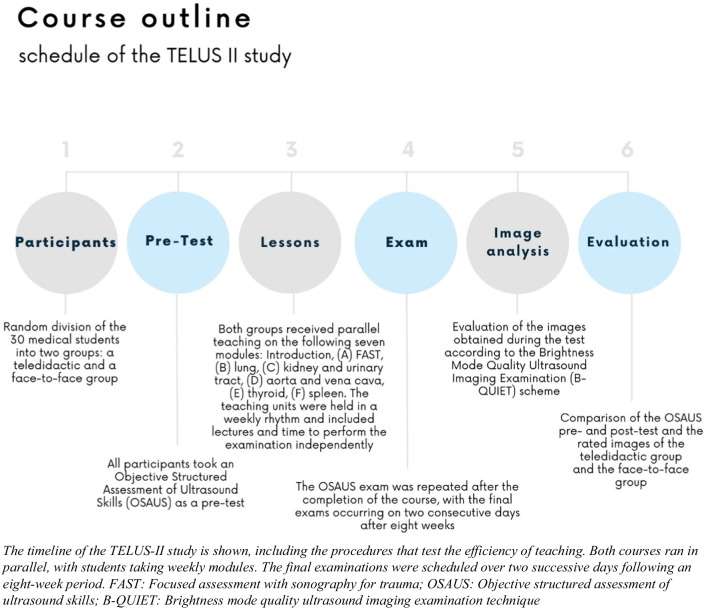
Timeline of the TELUS II study.

This study is a follow-up project and was supervised by two German Society for Ultrasound in Medicine (DEGUM) certified physicians (level I and level III). In the past, the course was already conducted as a proof of concept study using an online-only format (the TELUS I trial),^16^ as due to COVID restrictions, no hands-on teaching could take place with a control group. The aim of this study is to compare the learning success of teledidactic teaching with that of the face-to-face group that is now being provided. Participating students were in the clinical part of their medical studies and they could get credit for the ultrasound course as an elective subject. Within their degree program, students must select elective courses, allowing them the freedom to choose subjects based on their interests. The participating students have independently opted for this specific elective. Subsequently, they were randomly assigned to the teledidactic group and the face-to-face group (Fig. 1).

Each participating student received a mobile ButterflyIQ^17^ probe (Version 2, Butterfly Network Inc, Delaware, USA) for the duration of the course and, if necessary, an Apple iPad (Generation 9, Apple, Cupertino, USA) with the corresponding app to operate the probe.

### Course Outline

The course consisted of seven modules:

- (0) Introduction to ultrasound
- (A) Focused assessment with sonography for trauma (FAST)
- (B) Lung
- (C) Kidney and urinary tract
- (D) Aorta and vena cava
- (E) Thyroid gland
- (F) Spleen

**Figure 2:**
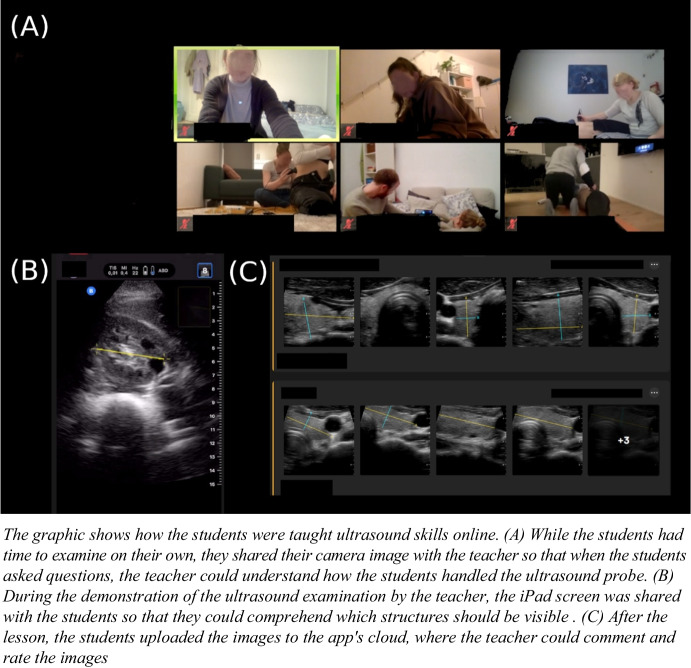
Demonstration of the implementation of the teledidactic ultrasound course.

The selection of the modules was based on the recommendations of an international consensus conference for undergraduate medical students.^18^ An introductory lecture was given at the beginning of each lesson. In the lecture, the guidance of the ultrasound probe during the examination was outlined. In addition, ultrasound images of typical pathologies were shown. In this way, the clinical reference was not missing, as the students examined each other or friends who were mostly young and healthy. The same slides were used online and face-to-face. After explaining the theoretical knowledge and guidance of the ultrasound probe, the teachers demonstrated the examination. During the online lessons, the camera image and the iPad screen of the teacher were shared simultaneously so that the students could follow the handling of the ultrasound probe (Fig. 2).

In the face-to-face course, the lecturer demonstrated the examination live with the help of a volunteer, who served as the model. After pending questions were clarified, the students had time to perform the examination on their own. In the online course, they had to organize a volunteer at home to perform the ultrasound examination, while the students assigned to the face-to-face course examined each other. During the lecture, the components that the students were required to include were thoroughly explained to them. For instance, they were told to take a longitudinal and cross-sectional slice of the kidney and measure its length, width, and thickness. While the students performed the examination on their own, the tutors provided direction and support. There were three tutors available for questions and corrections, so that approximately five students were supervised by one tutor. The instructor was able to correct students’ use of the ultrasound probe in the face-to-face course, as opposed to the online course, where assistance was only given in the form of words and videos. The captured ultrasound images were uploaded to the cloud using the Butterfly app. For the students from the teledidactic course to receive direct feedback on their images, the comment function of the Butterfly app was used and the teacher annotated the images to indicate how the presentation of the organs could be optimized. Direct feedback on images uploaded to the cloud was provided during the course upon student requests for clarification or when they faced challenges in recognizing organ structures. Furthermore, comprehensive feedback on these images, including specific comments, was delivered within the same week, ensuring timely responses prior to the subsequent module.

### Assessment Instruments

**Figure 3:**
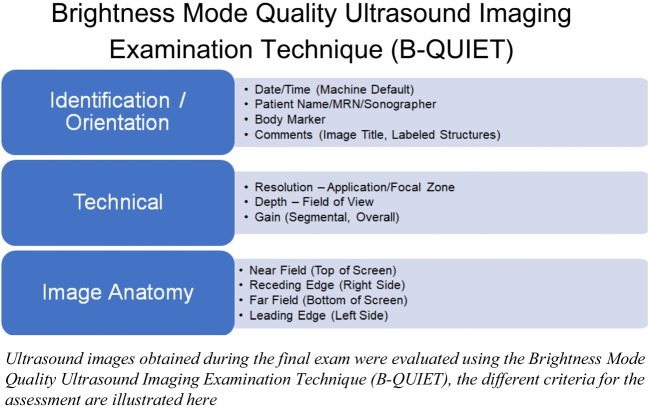
Evaluation criteria of the brightness mode quality ultrasound imaging examination technique (B-QUIET).

A suitable assessment tool had to be chosen to evaluate the students’ learning progress and compare the two groups. To test the various competencies, a combination of a practical test and an image rating was chosen. The Objective Structured Assessment of Ultrasound Skills (OSAUS)^19^ is an assessment tool explicitly designed for ultrasound examinations. The examination protocol can be used for the scanning of different organs and does not need to be specially adapted. OSAUS contains seven fields of evaluation, namely: indication for the examination, applied knowledge of ultrasound equipment, image optimization, systematic examination, interpretation of images, documentation of examination, and medical decision-making. Since a young, healthy patient was selected as the examination model, the scale had to be adapted. For instance, in the field medical decision-making of the thyroid gland, the keyword thyroid inferno was mentioned and asked in which disease this would occur, or in the vena cava examination, reasons for congestion of the vein had to be listed. Before the course, an OSAUS pre-test was conducted with the students to determine their level of knowledge and after completion of the course, the OSAUS exam was repeated to have a direct comparison. In the pre-test, not all modules were covered, but the protocol was limited to the FAST examination, the kidney, urinary tract, and thyroid gland. On the final exam, all the modules that were taught were tested, with the examination of the spleen integrated into the FAST examination, as the recessus splenorenalis had to be visualized. Various fabricated patient case scenarios served as the exam’s guides so that a clinical environment could be simulated. The students had a total of 25 min for each exam, giving them 5 min for each task to demonstrate the inspection of the corresponding organ. During the exam, every student, whether in a face-to-face or teledidactic group, scanned the same person. As a result, the test circumstances were consistent, making it impossible for the person’s prior practice with the test to have an impact. Using the brightness mode quality ultrasound imaging examination (B-QUIET) scale, ultrasound pictures from the final exam were evaluated.^20^ B-QUIET is a scale for ultrasound image evaluation and contains, among others, the item’s gain, depth, and resolution (Fig. 3). Three independent evaluators, consisting of two physicians and a peer tutor, conducted the lessons, assessed the images, and administered the pre- and post-OSAUS examination.

The OSAUS and image rating results of the face-to-face group were compared with those of the teledidactic group to determine whether online teaching can compete with the traditional way of teaching.

### Statistical Analysis

The statistical analysis was realized with RStudio (version 2022.07.1 + 554) and IBM SPSS Statistics 28. Means and standard deviations were calculated as descriptive parameters. Differences were found to be statistically significant when *p* < 0.05. A Levene’s test was performed to test for equality of variance followed by an independent *t*-test or single-factor ANOVA to test the null hypothesis indicating that there is no difference between the two groups. Using the B-QUIET scheme, a total of 450 images that were generated in the examination were rated by three independent raters, who also conducted the final OSAUS exam with the students.

The local ethics committee of the university approved the study and all enrolled students gave written informed consent to the participation in the course and to the use of their images. For managing incidental findings, we have utilized an article that provides a framework for defining and handling them within the context of ultrasound courses.^21^

## RESULTS

A total of thirty students took part in the study and were randomly assigned to a teledidactic and a face-to-face group. Though randomly assigned, the teledidactic group was comprised of more advanced students: three third-year, five fourth-year, and seven fifth-year students. In contrast, the face-to-face group consisted of six third-year, six fourth-year, and three fifth-year students.

### Objective Structured Assessment of Ultrasound Skills (OSAUS) Results

In the OSAUS pre-test, the face-to-face group achieved an average of 11.91 (SD ± 2.59) out of 35 points, whereas the teledidactic group achieved an average of 13.52 (SD ± 3.92) points and thus 1.61 points more (4.57%). On the final exam, students in the face-to-face group scored an average of 29.11 (SD ± 5.16) points out of 35 (83.17 %) and accordingly recorded an improvement of 17.2 points (49.14%) on average. Participating students of the teledidactic group scored an average of 30.54 (SD ± 2.73) points (87.2%) on the final exam, which is an improvement of about 17.02 points per student (48.63%). As a result, students in the teledidactic group scored 1.43 points higher on the final exam than students in the face-to-face group, corresponding to their advantage on the pre-test (Table 1).

**Table 1:** Results of the OSAUS Pre-test and Exam

Module	Pre-test teledidactic group	Final exam teledidactic group	Pre-test face-to-face group	Final exam face-to-face group	*P*-value regarding the final exam
FAST (A)	13.14 ± 4.29	31.15 ± 2.74	12.93 ± 4.53	29.73 ± 5.68	0.361
Lung (B)		30.31 ± 2.60		29.07 ± 5.80	0.340
Kidney (C)	13.64 ± 3.08	30.08 ± 2.77	11.93 ± 2.79	28.60 ± 4.31	0.260
Aorta and vena cava (D)		30.04 ± 2.68		29.13 ± 5.00	0.50
Thyroid gland (E)	13.79 ± 4.49	31.12 ± 2.85	10.87 ± 2.75	29.00 ± 5.54	0.164
Mean	**13.52** ± 3.92	**30.54** ± 2.73	**11.91** ± 2.59	**29.11** ± 5.16	

The results of the pre-test and the exam are displayed and the scores of the teledidactic group can be compared with those of the face-to-face group.

*OSAUS* objective structured assessment of ultrasound skills

**Figure 4:**
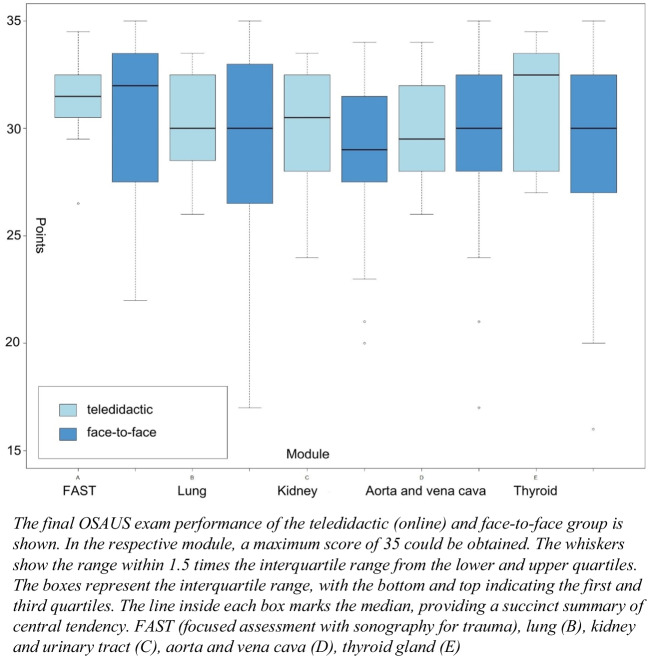
Differences in the OSAUS results for the organ-specific modules after teledidactic learning compared to traditional face-to-face teaching.

Both groups achieved the highest score in the demonstration of the FAST examination (teledidactic 31.15 ± 2.74, face-to-face 29.73 ± 5.68) (Fig. 4).

The peer tutor gave an average of 5 points out of a total of 175 possible points (5 modules of 35 points each) less than the experienced physicians. The three different OSAUS raters showed interrater reliability of 0.754 (95% confidence interval 0.587 < ICC < 0.852). Levene’s test indicated that there was no equality of variance between the two samples; therefore, a single-factor variance analysis was used for the investigation. The one-way ANOVA analysis reveals no significant difference between the two groups regarding the final exam (thyroid *p* 0.164, aorta and vena cava *p* 0.5, kidney and urinary tract *p* 0.260, lung *p* 0.340, FAST *p* 0.361).

### Image Analysis Results Using B-QUIET

The images taken in the pre-test were not used for evaluation. So, the image rating according to the B-QUIET scheme refers exclusively to images acquired in the final exam. The students who were assigned to the face-to-face group achieved an average of 33.93 (SD ± 3.71, 77.11 %) out of a possible 44 points. Students in the teledidactic group scored an overall mean of 35.43 (SD ± 3.19, 80.52%) points per US image; they, therefore, scored 1.5 points more on average, which corresponds to a difference of 3.4% out of a possible score of 44 points (Table 2). Considering the categories of resolution, depth, and gain, which are included in the B-QUIET scheme among others, the students lost points, especially when adjusting the depth correctly. Students in the face-to-face group received an average of 2.01 (SD 0.87) points out of a possible four in the depth category, compared to 2.9 (SD 0.77) points in gain and 2.85 (SD 0.80) points in resolution. Similarly, students in the teledidactic group only obtained 2.06 (SD 1.22) points in the depth category, 2.94 (SD 0.88) points in resolution, and 2.88 (SD 0.76) points in adjusting the gain. The *t*-test for independent samples shows that there is a significant difference in the FAST (*p* 0.015) and aorta (*p* 0.017) modules. In both modules, students in the teledidactic group performed better, scoring 33.59 (± 2.61) in FAST, whereas students in the face-to-face group scored an average of 30.95 (± 1.76). In the aorta module, the teledidactic group scored 35.41 (± 2.61) points, and students assigned to the face-to-face group, in comparison only 32.35 (± 3.08). Similar to the OSAUS exam results, no significant difference was found between the teledidactic and face-to-face group for the other modules (lung (B) *p* 0.225, kidney and urinary tract (C) *p* 0.160, thyroid (E) *p* 0.160).

**Table 2:** Results of the Image Rating Using the B-QUIET Method

Module	Face-to-face	Teledidactic	*P*-value
FAST (A)	30.95 ± 1.76	33.59 ± 2.87	0.015
Lung (B)	35.20 ± 3.33	33.42 ± 2.61	0.225
Kidney and urinary tract (C)	34.25 ± 2.11	35.62 ± 2.39	0.160
Aorta and vena cava (D)	32.35 ± 3.08	35.41 ± 2.61	0.017
Thyroid (E)	36.90 ± 4.75	39.09 ± 2.12	0.160
Mean	33.93 ± 3.71	35.43 ± 3.19	

The table reflects the results of the image evaluation, using the B-QUIET scheme. The students in the teledidactic course scored 1.5 points more on average, which corresponds to a difference of 3.4% out of a possible score of 44 points.

*FAST* focused assessment with sonography for trauma, *B-QUIET* brightness mode quality ultrasound imaging examination technique

## DISCUSSION

Our study demonstrates that teledidactic ultrasound teaching of the abdomen, thorax, and thyroid gland is comparable to traditional face-to-face teaching. The previous study that has been conducted by us should serve as a proof of concept,^16^ the control group now available is intended to validate the approach. A study for ocular ultrasound examinations has shown that online training was as good as hands-on training,^22^ as the groups did not differ in scan time and assessment scores, reflecting our findings in the OSAUS examination. An anatomy graduate course in ultrasound imaging^23^ and a study comparing point-of-care ultrasound training^24^ likewise showed no statistically significant differences in the assessment scores of students in the two groups, suggesting that ultrasound imaging can be taught to students without face-to-face teaching. A study examining the effectiveness and satisfaction of online teaching across ten different parameters, using a 5-point Likert scale, found that e-learning was more or equally effective in certain aspects, such as task delivery and meeting individual needs, but less effective in building skills and knowledge.^25^ The authors contend that, while technology cannot replace in-person instruction entirely, online instruction can help medical schools’ teaching efforts in some ways. However, in our study, the results of the teledidactic training did not differ from the face-to-face group in terms of improvement of their ultrasonography skills compared to the pre-test; the teledidactic group was equally successful in developing skills and knowledge.

Our study has several limitations, notably the relatively small sample size, since the group size was dictated by the number of ultrasound devices available, so it is possible that a small or moderate difference between the groups could have been missed. Figure 4 reveals significant deviations in modules B and E within the face-to-face group. These variances are largely due to the performance of three individuals who scored considerably lower than their peers. Specifically, some scored ten or more points below the group average in a total of only 35 points. Such disparities would likely be less pronounced in a larger sample size*.* Furthermore, the course participants either examined each other (face-to-face group) or a volunteer from their environment (teledidactic group), so that usually young, healthy people were examined on whom no pathologies could be demonstrated. Therefore, typical pathologies were discussed in the introductory lecture to provide a clinical reference, but it might be difficult for the students to identify them in real patients once the course is over. Because the program only lasted 8 weeks, no conclusions can be drawn about long-term outcomes. The two study groups were randomly divided; however, the teledidactic group contained more students in advanced years of study who may have had more clinical expertise at that time, possibly contributing to the superior performance in generating the images. Additionally, all of the students kept their ultrasound equipment accessible at home, so the effect of autonomous practice should not be overlooked, and it remains unclear whether there was any interaction between students across the two groups.

To determine precisely how online teaching should be carried out, further research should be done in the future. Standards should be created to establish the format of the skills testing in addition to the course framework. The incorporation of e-learning can enhance the effectiveness of the preparatory phase, potentially resulting in time savings during the face-to-face course phase.^26^ Therefore, a strategic decision should be made regarding the optimal balance between online and hands-on instruction based on the available resources and the desired outcomes of the educational program. Training medical students in the use of this radiation-free, widely used imaging device would be possible with minimal effort and at a reasonable cost. In underdeveloped areas, all that is needed to receive training is a portable ultrasound device, which is less expensive than conventional systems. Therefore, online concepts can be a valuable tool for medical students, as well as for the training and further education of residents and specialists. It is still difficult to find the most effective teaching method; however, it is clear that new methods need to be developed and used.

## CONCLUSION

This pilot study has shown that the effectiveness of teledidactic training in basic abdominal, thoracic, and thyroid ultrasound appears similar to hands-on training, suggesting that this may be a useful teaching approach during times like the COVID-19 pandemic. In two modules of the assessed ultrasound images, the teledidactic group performed statistically significantly better than the face-to-face group, indicating that online teaching should strongly be considered in curriculum development. The digital adoption of a portable, cost-effective point-of-care ultrasound technology may present a significant chance to improve ultrasound training on a global scale.
